# Soldiers in the Provincial Hospitals

**Published:** 1914-09-26

**Authors:** 


### SOLDIERS IN THE PROVINCIAL HOSPITALS.

(FROM OUR SPECIAL CORRESPONDENTS.)

### ASHTON-UNDER-LYNE: ANTI-TYPHOID INOCULATION.

The governors of the Ashton-under-Lyne Infirmary have been informed by the British Red Cross Society that the military authorities have accepted the offer of the board to set aside twenty beds for wounded sailors or soldiers during the war. By a rearrangement of the wards it will be possible to do this without decreasing the number of beds normally available for the civil population, although a considerable sum will have to be expended on additional equipment. The governors also have consented to place the out-patient department at the disposal of the War Office for the treatment with anti-typhoid vaccine of the troops stationed at Ashton Barracks. The honorary staff, the resident doctors, and members of the nursing staff will all help in this work, which will be carried out at such times as not to interfere with the ordinary use of the department.

This infirmary has already been the means of adding several men who required operative treatment to the fighting strength of the Army, and many others are now either under treatment or upon the "waiting list." In the out-patient and casualty department several injured soldiers sent back from the fighting line to their depôts have received necessary surgical attention.

### BRADFORD.

At the outbreak of hostilities the Bradford Branch of the British Medical Association offered to give gratuitous medical attendance to the dependants of naval men and Regular and Territorial troops, and this scheme has been in operation for some weeks. In many instances free medicines have also been supplied by the doctors. The Bradford Royal Infirmary has lost another resident officer, Dr. C. B. Marshall, M.D., D.P.H., who has been called up for duty with the Manchester Infantry Brigade. He was appointed resident surgical officer *vice* Mr. F. W. Nunneley, M.B., who received his commission in the Naval Medical Service. There has been a ready response to Earl Kitchener's appeal for blankets for the troops, two large bales, comprising about 200 blankets in all, having already been despatched to York. No wounded soldiers have been sent to Bradford as yet. The Lord Mayor's fund for the relief of distress arising from the war now amounts to £21,000, and is steadily increasing. A committee has been formed to assist Belgian refugees, and a house has been placed at their disposal. A local fund for this purpose has been inaugurated.

### BRISTOL: SUPPLEMENTARY HOSPITAL.

The Royal Infirmary, Bristol, has acquired an excellent and valuable supplementary hospital in "Bishop's Knoll," a picturesque mansion which was placed at the disposal of the War Office at the outbreak of the war by Mr. R. E. Bush. The necessary equipping of the building as a hospital has been going forward, and at the time of writing 100 wounded can be accommodated, and about twenty men are understood already to be occupying beds.

### CAMBRIDGE: NEW QUARTERS OF THE 1st EASTERN GENERAL HOSPITAL.

A very large proportion of the wounded soldiers who were admitted and have been under treatment at the R.A.M.C. (T.) 1st Eastern General Hospital have recovered sufficiently to leave the hospital. They received of the best which medical, surgical, and nursing science can offer, and they were not lacking in their expressions of gratitude. To a man they craved for a speedy and complete recovery to enable them to return to the seat of war. Many ladies and gentlemen in Cambridge and the neighbouring districts have sent to the 1st Eastern General Hospital for the benefit of the sick and wounded gifts of flowers, fruit, vegetables, wine, game, eggs, honey, jam, clothing, pipes and tobacco, newspapers and magazines. The Soldiers' and Sailors' Families' Association Fund now amounts to over £4,000. The appeal of Lord Kitchener has not fallen on deaf ears, for a large number of young men have enlisted and are undergoing complete training in Cambridge, which is now considered a military centre for training. Through the efforts of Dr. C. F. Searle the 1st East Anglian Field Ambulance Corps has been considerably strengthened by the enlistment of a large number of Cambridge young men as well as undergraduates from St. John's and Trinity Colleges.

The Cloisters and Neville's Court at Trinity College, Cambridge, in their converted state, which now forms the 1st Eastern General Hospital, have to be vacated early in October, practically as soon as term commences. In view of the fact arrangements are being made for providing hospital accommodation for at least 500 patients by erecting a suitable temporary hospital on Clare College Cricket Ground, Burrell's Walk, Cambridge. Some of the wounded soldiers are still under treatment at the hospital, and are progressing favourably. During the time the Cloisters at Trinity College have been used as a hospital for the sick and wounded soldiers the accommodation has been supplemented by the erection of tents. On Sunday morning, September 13, Herbert Francis Paty, Second Lieutenant, 1st Battalion North Staffordshire Regiment, who occupied one of these tents, was found shot dead. At the inquest held on Monday, the following day, by the Borough Coroner a verdict was given that the deceased shot himself, but there was not sufficient evidence to show the state of his mind at the time.

The following members of the staff at Addenbrooke's Hospital, Cambridge, are now on active military service: Mr. H. F. Rutherford, in-patient and stores clerk; Mr. C. Charter, senior dispensary apprentice; Mr. F. Duke, attendant clinical laboratory, R.A.M.C., 1st Eastern General Hospital.

Mr. A. R. Dyball, out-patient clerk, has responded to the appeal and has enlisted in Lord Kitchener's Second Army. In all such cases the appointment will be kept open. The vacancies caused by these responses to the country's call are only being temporarily filled.

Dr. Dalton Mallam, house surgeon at Addenbrooke's Hospital, and Dr. E. J. Y. Brash, second house surgeon, have received commissions in the Royal Army Medical Corps (T.), with rank of captain, and are both on duty at the 1st Eastern General Hospital. Dr. S. A. Lane, house physician, has also received a commission in the R.A.M.C. and has volunteered for foreign service. Cambridge has not been wanting in offering shelter not only to Belgian, but to French refugees.

Members of the Cambridge Branch of the Red Cross Society continue to have opportunities of gaining practical experience by attending the Cambridge Workhouse Infirmary and at the casualty department of Addenbrooke's Hospital. They also have the privilege of witnessing minor operations and attending in the wards. Dr. Haynes, Dr. Bowen, and Mr. Kempson continue to lecture to the members. Dr. Charles H. Budd, late house physician of St. Thomas's and Addenbrooke's Hospitals, is also delivering a course of lectures. In addition to these lectures Miss Somerfield, sister of the Victoria Ward, Addenbrooke's Hospital, has also delivered a course.

The Red Cross Society has been most successful—in addition to raising funds—in obtaining a good supply of clothing and other useful articles not only for the benefit of the wounded whilst in hospital, but for their benefit on discharge.

A number of ladies and gentlemen both in the county and town of Cambridge are actively engaged in organising concerts, the proceeds of which are to be given either to the Red Cross Society, the Prince of Wales' Fund, or to the Soldiers' and Sailors' Families' Association Fund.

Baron von Hügel, of Croft Cottage, Cambridge, has been successful in his appeal on behalf of Belgian refugees. A home large enough to accommodate two families is now ready.

### GRAVESEND AND DISTRICT.

No wounded have yet arrived at the Gravesend Hospital, but several soldiers from the barracks and forts in the district have been treated for small ailments and slight accidents, and others have attended for $x$-ray diagnosis. There are three or four soldiers in the wards from various regiments who have had operations, and one who has been medically treated states he was present at Mons with the Royal Horse Artillery. He is a native of Chalk, a village near by, and was on his way home from the Royal Herbert Hospital, where he has been invalided, when he was ordered to the hospital.

The first meeting of the enlarged executive committee of the Gravesend and Northfleet Voluntary Aid Detachments was held at the headquarters on Thursday, September 17. Sir Gilbert Parker, M.P., presided, and was supported by Alderman Goldsmith, Councillor H. E. Porter, and Mr. H. D. Stephenson, chairman of the hospital. Sir Gilbert Parker announced that Lady Parker had made appeals for subscriptions from friends outside Gravesend and Northfleet to be used in the purchase of hospital beds and their full equipment, with the very gratifying result that he was able to hand over to the treasurer that evening Lady Parker's cheque for £150 towards that object. A report by the Repairs and Works Sub-Committee showed that a steady progress was maintained in the work of transforming the Yacht Club into a hospital, and that that work was now nearing completion. Mr. H. L. Tatham was offered and accepted the office of commandant of the men's detachment, which is now in course of formation, and Dr. Drennan was appointed medical officer. It was stated there was good reason to believe that the War Office will, before long, avail itself of the services of local detachments, and when the call comes it will find them fully prepared.

### LEICESTER: FURTHER WOUNDED ARRIVE.

ADVICE was received last Saturday at the 5th Northern General Hospital that a further batch of wounded would be sent, and on Sunday afternoon at 4.15 an ambulance train from Southampton, in charge of Captain Stracey, steamed into the Midland station with 125 wounded men. Under the supervision of Mr. A. W. Faire, County Director, Voluntary Aid Detachments, a fleet of sixty motors was waiting to transport the men to their quarters. The route to the hospital was lined with spectators, and vociferous cheers greeted the first motor-car as it sped on its way from the station with the helmet of a German officer proudly displayed in front. As on the former occasion, it took just about half an hour for the transport of the men to the hospital where everything was in readiness for them. The base hospital, in fact, is working with a smoothness and ease which only comes with efficient administration, and the arrangements made by Colonel Harrison and his staff for the disposition of the men on arrival showed that much attention had been given to details.

The wounded men—who come from Mons, Soissons, Compiègne, and the Aisne—had already received treatment in the base hospital at St. Nazaire, on the banks of the Loire, and they spoke most gratefully of the treatment they received there. Some few of them were severely injured, others had had amputations, but most of them were not seriously wounded, and it is hoped with a fortnight's treatment and care many of these will be able to return to their depôts. Of the first batch of wounded still remaining all are making good progress, and it seemed quite like pre-war times to see them playing the game of the "muddied oafs" in the playing-field of the hospital. During the week Captain T. C. Clare has been mobilised for duty in charge of the pathological and bacteriological departments. Congratulations are due to Lieut.-Col. L. K. Harrison on his recent preferment, to date from the mobilisation of the hospital. Under the direction of the Rev. W. Cyril Luxmoore, the hospital chaplain, a series of entertainments has been arranged for Tuesday and Saturday evenings.

### LINCOLN: 110 WOUNDED RECEIVED.

The 4th Northern General Hospital (Territorial) is now occupied by 420 patients. One hundred and ten of these are wounded men from the Front; the rest are sick. The hospital is now in full working order and ready to receive its full number of patients, which forms a complement of 520.

### SHEFFIELD: EMBARRASSING SYMPATHISERS.

Of the 125 wounded soldiers brought to the 3rd Northern General Hospital, installed in Sheffield Teachers' Training College, at the beginning of the month, only thirty-four now remain, the others having been discharged either on furlough or returned to duty. Many of these were comparatively slight cases—quite a number, in fact, were suffering from nothing more serious than the effects of the long forced marches, for which they were ill-prepared physically. In the expectation of receiving a larger number of more serious cases in the next convoy, the commanding officer has sent in a return of a considerable number of vacant beds. With the neighbouring hospitals at Leeds and Lincoln fully occupied, the Sheffield authorities are keenly anxious that the provision they have made shall be put to greater use than is at present the case. Sheffield people generally have made the wounded soldiers very welcome. Gifts of game, fruit, tobacco, special articles of clothing asked for, and many other creature comforts have been showered upon them, while offers of entertainment have been so numerous that opportunity has not yet been found even to acknowledge them. As the longer evenings approach, however, and with the prospect of a larger number of convalescents, these offers will doubtless prove very welcome. In one other respect the attentions of the Sheffield sympathisers have proved almost embarrassing to the hospital authorities. The convalescents have been taking advantage of the recent fine weather to take their exercise in the spacious grounds attached to the Training College, which are divided from the road only by a low wall, and over this the soldiers have been in the habit of chatting with their new-found friends, and these latter have assembled in such numbers that the authorities have found it necessary to barricade the road and erect a wooden fence above the wall.

